# Utilizing high-throughput experimentation to enhance specific productivity of an *E.coli *T7 expression system by phosphate limitation

**DOI:** 10.1186/1472-6750-11-22

**Published:** 2011-03-17

**Authors:** Robert Huber, Simon Roth, Natalie Rahmen, Jochen Büchs

**Affiliations:** 1 AVT - Aachener Verfahrenstechnik, Biochemical Engineering, RWTH Aachen University, Worringerweg 1, D-52074 Aachen, Germany

## Abstract

**Background:**

The specific productivity of cultivation processes can be optimized, amongst others, by using genetic engineering of strains, choice of suitable host/vector systems or process optimization (e.g. choosing the right induction time). A further possibility is to reduce biomass buildup in favor of an enhanced product formation, e.g. by limiting secondary substrates in the medium, such as phosphate. However, with conventional techniques (e.g. small scale cultivations in shake flasks), it is very tedious to establish optimal conditions for cell growth and protein expression, as the start of protein expression (induction time) and the degree of phosphate limitation have to be determined in numerous concerted, manually conducted experiments.

**Results:**

We investigated the effect of different induction times and a concurrent phosphate limitation on the specific productivity of the T7 expression system *E.coli *BL21(DE3) pRhotHi-2-EcFbFP, which produces the model fluorescence protein EcFbFP upon induction. Therefore, specific online-monitoring tools for small scale cultivations (RAMOS, BioLector) as well as a novel cultivation platform (Robo-Lector) were used for rapid process optimization. The RAMOS system monitored the oxygen transfer rate in shake flasks, whereas the BioLector device allowed to monitor microbial growth and the production of EcFbFP in microtiter plates. The Robo-Lector is a combination of a BioLector and a pipetting robot and can conduct high-throughput experiments fully automated. By using these tools, it was possible to determine the optimal induction time and to increase the specific productivity for EcFbFP from 22% (for unlimited conditions) to 31% of total protein content of the *E.coli *cells via a phosphate limitation.

**Conclusions:**

The results revealed that a phosphate limitation at the right induction time was suitable to redirect the available cellular resources during cultivation to protein expression rather than in biomass production. To our knowledge, such an effect was shown for the first time for an IPTG-inducible expression system. Finally, this finding and the utilization of the introduced high-throughput experimentation approach could help to find new targets to further enhance the production capacity of recombinant *E.coli*-strains.

## Background

One of the major goals in biological process development is to increase the productivity of fermentation processes, in particular, to increase the amount of protein produced per cell (specific productivity). Advantages thereof are usually less effort for product purification, lower fermentation volumes and, thus, smaller bioreactors that are needed to produce a certain amount of product. This can lead to significant cost reductions. The specific productivity can be influenced by genetic engineering of strains, choice of the host/vector system (e.g. plasmid copy number and promoter), media optimization or process optimization (e.g. optimizing inducer concentration and induction time; reducing acetate formation by using fed-batch mode) [1]. In particular, the induction time is probably the most crucial factor for inducible recombinant expression systems [2]. Therefore, it is important to screen different induction times, especially if other means of increasing productivity should be evaluated.

Another widespread concept to increase specific productivity is to decouple biomass build-up from product formation [3-5]. When recombinant proteins are produced in growing cells, energy and nutritional resources are often directed toward biomass production rather than target protein formation [6]. To circumvent this, the idea is to use cells that are non-growing (dormant) yet are metabolically active and capable of producing protein at higher levels. Rowe and Summers designed such a system (termed Quiescent Cells) using a genetically modified *E.coli *strain that provides a higher specific productivity by decreasing chromosomal gene expression (and, hence, growth) in favor of plasmid-encoded gene expression [6-8]. Another concept is to induce protein expression in the stationary phase of a batch culture [4-6]. Here, the gene of interest is placed under the control of a starvation-inducible promoter that is turned on when glucose becomes exhausted [9]. Compared with exponentially growing cells, stationary phase cells are far more resistent to stress [10,11] and might therefore be suited to produce recombinant proteins [12], as this can place a huge metabolic burden on the cells [13,14]. Another starvation-inducible promoter is based on the alkaline phosphatase (*phoA) *gene. When phosphate is depleted in the medium, the target genes under the control of the *phoA *promoter are induced [15,16]. This system is described for the expression of a number of recombinant proteins [9,15,17,18]. A further possibility to limit growth and hereby enhance product formation is to limit secondary substrates such as magnesium, potassium or especially phosphate in the cultivation medium [19].

Phosphate - being one of the most abundant elements in *E.coli *in terms of the cellular content - plays a crucial role in the cell. Wanner summarized many aspects of the role of phosphate on cell's structure and function [16]. This includes the incorporation of inorganic phosphate in membrane lipids, nucleic acids, carbohydrates and other cellular metabolites. Here, phosphate also acts as an effector of enzymatic reactions [20] and is thus involved in many metabolic pathways, especially in the energy metabolism of the cell (e.g. as high-energy phosphoanhydride bond in ATP). Furthermore, polyphosphate acts as an energy- and phosphate-storing polymer that is used during phosphate limitation [16,21]. Because of this universal role of phosphate, a limitation of this nutrient has tremendous effects on the cell growth and physiology. Therefore, many genes are found to be regulated by the level of inorganic phosphate in the medium [22]. A phosphate exhaustion in an *E.coli *cultivation leads to activation of the Pho regulon [16]. Furthermore, the RpoS response is triggered [23] and cells enter the stationary phase [11]. Due to phosphate limitation, the expression of hundreds of proteins is induced and, at the same time, the levels of more than two hundred proteins are reduced [10,24]. In summary, a phosphate limitation leads to quite complex regulatory mechanisms in the cell and is still not yet fully understood on the molecular level.

Nevertheless, phosphate exhaustion has been shown to bolster product formation. This was recently proved in batch cultures for a catabolite-repressed *Hansensula polymorpha *strain with glucose or glycerol as carbon source [19]. Jensen and Carlsen cultivated an *E.coli *strain with an constitutive promoter for a recombinant protein in fed-batch mode. By limiting phosphate in the medium they were able to stop the biomass build-up and enhance specific productivity [25]. A similar effect could be achieved for producing shikimic acid in *E.coli *in a phosphate-limited chemostat culture [26] and for lysine production with *Corynebacterium glutamicum *[27]. However, besides using the *phoA *promoter, there are only few reports on phosphate-limited batch cultivations of *E. coli *[26]. Moreover, there are no reports specifically on using phosphate limitation with the most common inducible promoter system, i.e. the T7 or *lac *promoter, to enhance specific productivity of recombinant protein expression. The reason for this might be the difficulty to establish optimal conditions for inducing protein expression in phosphate-limited cultures. For this purpose, many experiments have to be conducted in parallel batch cultures to determine the best phosphate content of the medium and the optimal time of induction. So far, such studies are very tedious with conventional means (e.g. shake flask or microtiter plates (MTPs) experiments with offline-measurement of growth and manual induction of protein expression at an appropiate time).

Hence, the aim of this work was to investigate different induction times and in particular to increase specific productivity of a T7 expression system via phosphate limitation. Therefore, the influence of phosphate on growth and recombinant protein expression (at various induction times) in batch cultures was studied with specific online-monitoring tools for small scale cultivations, namely RAMOS [28,29] and BioLector [30,31]. Furthermore, a novel cultivation platform (termed Robo-Lector) was applied [32]. This system conducts high-throughput experiments in MTPs automatically and allows to monitor microbial growth and the production of fluorescence proteins online.

## Methods

### Microorganism

For all experiments the strain *E.coli *BL21(DE3) pRhotHi-2-EcFbFP was used (kindly provided by T. Drepper, Institute of Molecular Enzyme Technology, Heinrich-Heine-University Düsseldorf, Germany) [33]. The used expression plasmid harbors a kanamycin resistence gene and the T7 promoter that is under the control of the *lac *operator. The fluorescent protein EcFbFP is expressed as a model protein. Therefore, the EcFbFP encoding gene was cloned into the pRhotHi-2 vector downstream of the T7 promoter. A His_6_-tag was fused to the C-terminus of the EcFbFP resulting in a recombinant fusion protein with a molecular weight of 16.5 kDa. The used EcFbFP is also available under the trademark evoglow (evocatal GmbH, Düsseldorf, Germany).

### Media and Solutions

For all precultures TB-Medium was used, consisting of 24 g/L yeast extract, 12 g/L tryptone, 12.54 g/L K_2_HPO_4_, 2.31 g/L KH_2_PO_4 _and 5 g/L glycerol. The medium was supplemented with kanamycin (50 μg/L) and the pH was adjusted to 7.2 with NaOH.

All main cultivations were performed in a modified form of Wilms & Reuss synthetic medium [34,35]. The medium consists of 7.5 g/L glucose, 5 g/L (NH_4_)_2_SO_4_, 0.5 g/L NH_4_Cl, 3 g/L K_2_HPO_4 _(for phosphate unlimited conditions), 2 g/L Na_2_SO_4_, 0.5 g/L MgSO_4_•7H_2_O, 41.85 g/L 3-(N-Morpholino)-propanesulfonic acid (MOPS), 0.01 g/L thiamine hydrochloride, 1 mL/L trace element solutions. For determining the phosphate demand of the applied strain, different media were prepared with the following phosphate concentrations (as K_2_HPO_4 _in mg/L): 3000, 750, 300, 225, 195, 180, 150, 105, 90, 75, 60, 45, 30. All media preparations were supplemented with kanamycin (50 μg/L) and the pH was adjusted to 7.5 with NaOH.

For induction of protein expression a sterile-filtered IPTG stock solution (100 mM) was used to give a final IPTG concentration of 0.1 mM in the cultures. This concentration was identified in previous experiments to be suitable for efficient recombinant protein expression (data not shown). All reagents were of analytical grade and supplied by Carl Roth GmbH & Co. KG (Karlsruhe, Germany).

### Cultivation

#### Preculture

The precultures for all experiments were inoculated from a cryoculture of *E.coli *BL21(DE3) pRhotHi-2-EcFbFP and cultivated in a 250 mL Erlenmeyer shake flask with 10 mL TB-medium. The flask was placed on a rotary shaker at a shaking frequency (n) of 300 rpm, a shaking diameter (d_0_) of 50 mm and at a temperature (T) of 37°C for 16 h (t). To ensure that no disturbing media components from the preculture medium - especially phosphate - influence the limitation experiments, the harvested cells were washed twice. Therefore, cells of the preculture were pelleted by centrifugation at 4000 rpm and resuspended in phosphate-free modified Wilms & Reuss medium. After two washing steps, the subsequent main cultures were inoculated to give an initial optical density (OD) of 0.4. OD was measured at 600 nm with a Uvikon 922 spectrophotometer (Kontron, Milano, Italy).

#### Microtiter plate cultivations

**Figure 1:**
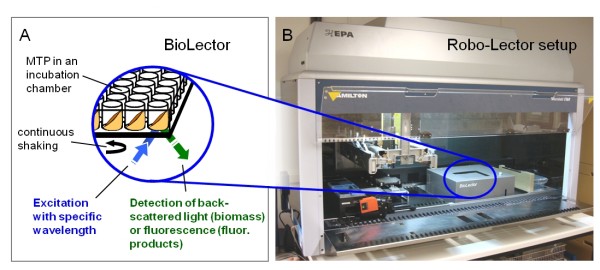
**Measurement principle of the BioLector (A) and experimental setup for the Robo-Lector (B)**. The BioLector provides a system for cultivating microorganisms in a microtiter plate (MTP) inside an incubation chamber. The MTP is continuously shaken to provide sufficient oxygen to the culture. An optical measurement system continuously monitors biomass concentration and fluorescent products. The Robo-Lector setup consists of a BioLector integrated into a pipetting robot, enabling fully automated high throughput cultivation experiments in a MTP format.

Cultivations in MTPs were conducted in a BioLector [30,31] which is a system that can permanently monitor microbial growth and the fluorescence of reporter proteins under defined conditions in a MTP during the cultivation. Therefore, a MTP with medium is inoculated and placed in the incubation chamber of the BioLector (Figure 1A). The MTP is continuously shaken to provide a sufficient oxygen transfer to the culture. For growth and product monitoring, light with a defined wavelength is sent into each well (excitation), whereas the backscattered light (indicator for biomass) or fluorescence (indicator for fluorescent products) is detected and analysed. In this study, the experiments to determine the phosphate demand and the influence of phosphate limitation on protein production were conducted in a BioLector prototype [30].

The screening for the optimal induction time was carried out with the Robo-Lector platform described by Huber et al. [32]. The Robo-Lector platform consists of a BioLector (m2p-labs, Aachen, Germany) [31] integrated into a pipetting robot (Figure 1B). This system enables one to conduct automated high-throughput cultivations. Here, the method 'induction profiling' was used, which performs an automated induction of parallel cultures by the pipetting of IPTG-solutions in the respective wells of a main culture MTP at different times. For a detailed description of the method please refer to Huber et al. (2009). The method was used with the following modifications: the induction time was varied from 1 to 10 h and the inducer concentration was kept constant at 0.1 mM. The following conditions were applied for all cultivations in the BioLector prototype and the Robo-Lector: 37°C, shaking diameter 3 mm, shaking frequency 950 rpm. All cultivations were performed in sterile black 96 well MTP (μClear, Cat. 655087, Greiner Bio-One, Frickenhausen, Germany) with an initial filling volume of 200 μL (BioLector prototype) or 190 μL (Robo-Lector). To avoid evaporation, each utilized MTP were sealed with a gas-permeable adhesive seal (ThermoScientific, Waltham, USA) for the cultivations in the BioLector prototype. For the Robo-Lector experiments, the MTP was sealed with sterile pierceable, resealable tape (X-Pierce, Excel Scientific, Victorville, USA), allowing ventilation of the wells at reduced evaporation rates. The EcFbFP fluorescence was monitored at an excitation wavelength of 460 nm and an emission wavelength of 492 nm. The resulting fluorescence is a suitable quantitative signal for *in-vivo *EcFbFP expression [36,37] and therefore corresponds to the total volumetric yield of the target protein EcFbFP. The biomass concentration was measured via scattered light intensity (I) and was detected at an excitation wavelength of 620 nm. The initial scattered light intensity (I_0_) was mainly attributed to factors such as the media background or the type of the MTP and thus was substracted from the residual scattered light data (I-I_0_) [30] or fluorescence data (where appropiate). Different signal gain factors for scattered light and fluorescence were used as stated in the figure captions. The specific productivity for EcFbFP expression in the MTP cultivations was defined as EcFbFP fluorescence per scattered light intensity (given in a.u.).

#### RAMOS cultivations

The RAMOS device provides analysis of microbial growth in shake flasks by measuring the respiratory activity of the microorganisms [28,29,38]. The respiration activity (Oxygen Transer Rate, OTR) in different phosphate-containing cultures was measured with the RAMOS device in modified 250 mL Erlenmeyer shake flasks with a filling volume of 10 mL. Shake flasks with 3000 mg/L, 150 mg/L and 75 mg/L K_2_HPO_4 _were cultured in duplicate. All cultivations were carried out with a shaking frequency of 350 rpm and at a shaking diameter of 50 mm on a Lab-Shaker LS-K (Kühner, Birsfelden, Switzerland) at T = 37°C. To determine PO_4_^3-^, glucose and acetate concentrations, separate shake flasks were cultured in a Lab-Shaker LS-K under identical conditions as in the RAMOS device. From these shake flasks samples were collected approximately every 3 h. Phosphate (PO_4_^3-^) was analysed using a photometric kit (Spectroquant, Cat. 1.00616.0001, Merck, Darmstadt, Germany). PO_4_^3- ^has a molar fraction of 55% in K_2_HPO_4_, hence 3000 mg/L K_2_HPO_4 _correspond to 1650 mg/L PO_4_^3-^, 150 mg/L K_2_HPO_4 _correspond to 83 mg/L PO_4_^3- ^and 75 mg/L K_2_HPO_4 _correspond to 41 mg/L PO_4_^3-^. Glucose and acetate were measured in duplicate via HPLC (Ultimate 3000, Dionex, Sunnyvale, USA) on an organic acid resin (CS-Chromatographie Service, Langerwehe, Germany) at 60°C with 5 mM H_2_SO_4 _as eluent.

#### Shake flask cultivations

Shake flask experiments were conducted to verify results from the MTP scale. These cultures were grown in 250 mL Erlenmeyer shake flasks which were filled with 10 mL modified Wilms & Reuss medium with different phosphate concentrations (3000, 225, 150, 75 mg/L K_2_HPO_4_) at a shaking frequency of 350 rpm, a shaking diameter of 50 mm and 37°C. The cultures were induced with IPTG (final concentration of 0.1 mM) after 6 h of growth. After 26 hours, the OD of the cultures was measured and cells were harvested to perform product analysis via SDS-PAGE. Furthermore, scattered light and fluorescence intensities of culture samples (200 μL) were measured in the BioLector prototype.

### SDS-PAGE and densitometry

SDS-PAGE was performed to correlate the specific productivities of the shake flask cultivations with different phosphate concentrations. The SDS-PAGE was conducted with the XCell SureLock MiniCell (Invitrogen, Carlsbad, USA) and a Bis-tris-gel (NuPAGE 4-12%, Invitrogen) according to the manufacturer's instructions. Normalized samples were prepared by suspending equal amounts of cells from the shake flask experiments in SDS-buffer and loading them on the gel. Additionally, a molecular weight standard (RotiMark, Roth, Crailsheim, Germany) was loaded on the gel. After electrophoresis, the gel was stained and destained according to the manufacturer's instructions (SimplyBlue SafeStain, Invitrogen). The destained gel was photographed with the documentation system ChemiDoc (Biorad, Hercules, USA) and analyzed with the densitometry computer program Total Lab TL100 (Nonlinear Dynamics, Newcastle upon Tyne, UK) to determine the specific productivity of the different samples. The specific productivity was defined as the ratio of the density of the target protein band (EcFbFP) to the total density of all bands of the corresponding sample (band%).

## Results and Discussion

### Effects of phosphate limitation on growth

**Figure 2:**
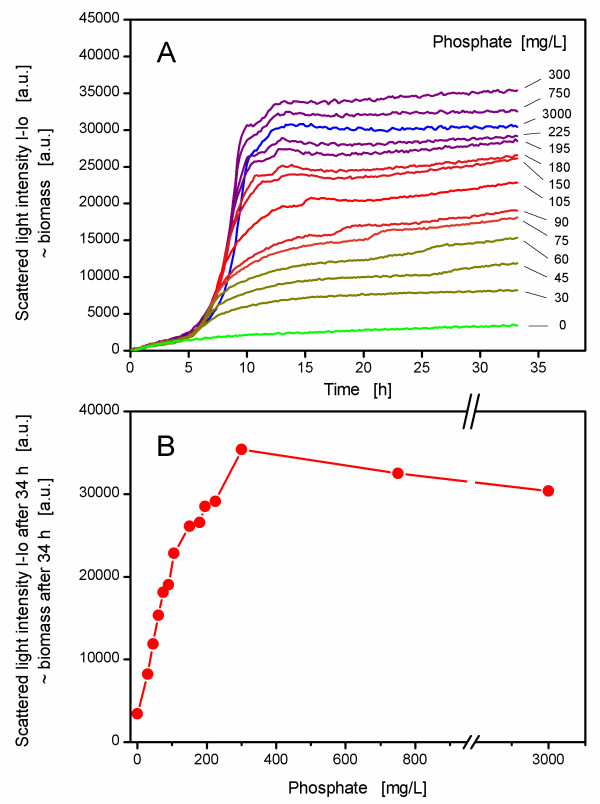
**Determination of phosphate demand of *E.coli *BL21(DE3) pRhotHi-2-EcFbFP via batch experiments in different media**. The colored curves in panel (A) represent the mean values (n = 6) of cultures grown in media (modified Wilms & Reuss medium with 7.5 g/L glucose) containing phosphate from zero to 3000 mg/L. The relative standard deviation of each curve did not exceed ± 10%. The cultivation conditions were as follows: 37°C, shaking diameter 3 mm, shaking frequency 950 rpm, filling volume 200 μL, no inducer was added. The measurements were conducted with the BioLector prototype with a signal gain factor of 30. Panel (B) depicts the biomass after 34 h of growth as a function of the applied phosphate concentration in the medium (data taken from experiment shown in panel A).

The aim of this study was to examine the effects of a phosphate limitation on the growth of the *E.coli *strain BL21(DE3) pRhotHi-2-EcFbFP and its recombinant product formation. First of all, the growth behavior at different phosphate concentrations was investigated in the BioLector device. A graduated growth pattern resulted from this experiment (Figure 2A). When no phosphate was added to the medium (0 mg/L) there was nevertheless some growth visible. This might be explained by some residual phosphate from the preculture (though these cells were washed two times with phosphate-free medium) and most likely because of intracellular polyphosphate stores which were mobilized during phosphate starvation [11]. The phosphate content of the original medium was 3000 mg/L, and this culture showed a typical batch culture curve with exponential growth. The stationary phase began at approximately 13 h with a biomass concentration of 30000 a.u. (Figure 2A). This culture was phosphate unlimited, whereas the cultures with 300 and 750 mg/L reached a slightly higher scattered light intensity. This could be explained by the fact that excess phosphate in the medium (3000 mg/L) could form a complex with trace elements (unpublished results) and this, in turn, led to a slightly lower biomass yield compared to 300 or 750 mg/L phosphate. The cultures with less than 300 mg/L phosphate showed lower biomass yields as phosphate became more and more limited. This correlation can also be seen in Figure 2B, where an almost linear relationship between biomass yield (scattered light intensity at the end of the cultivation) and phosphate content of the medium becomes apparent. From such a depiction, the minimal phosphate demand of the cells for unlimited growth (here: 300 mg/L) could easily be determined [39]. All cultures with phosphate showed the same increase in scattered light intensities in the beginning (Figure 2A), indicating a comparable maximum specific growth rate (μ_max _= 0.79 ± 0.09). As soon as phosphate became limited, these cultures deviated from the unlimited cultures and then continued with slow growth until they reached a plateau (Figure 2A). This slight increase in biomass concentration had been shown elsewhere [11,18,40] and seemed typical because of the metabolism of intercellular phosphate reserves. With the approach to monitor growth in MTPs, the demand of the examined *E.coli *strain for phosphate could be investigated in detail. Futhermore, the time when phosphate became limited was derived from the online-monitored growth curves.

**Figure 3:**
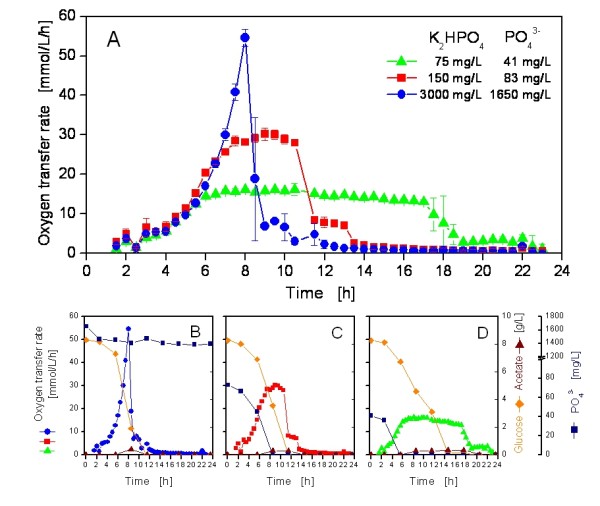
**Influence of phosphate limitation on respiration activity during cultivation**. The oxygen transfer rate (OTR) was measured with the RAMOS device. In panel (A), the mean (n = 2) and standard deviations of duplicate cultivations with 3000 mg/L K_2_HPO_4 _(•, blue), 150 mg/L K_2_HPO_4 _(■, red) and 75 mg/L K_2_HPO_4 _(▲, green) are depicted. In separate shake flasks, samples were collected and analysed for glucose, acetate and phosphate (PO_4_^3-^). The results of these analyses and the OTR are depicted in (B) for 3000 mg/L, in (C) for 150 mg/L and in (D) for 75 mg/L K_2_HPO_4_. The cultivation conditions were as follows: 37°C, shaking diameter 50 mm, shaking frequency 350 rpm, filling volume 10 mL, no induction; modified Wilms & Reuss medium (7.5 g/L Glucose) with various phosphate contents.

In order to verify these results, a cultivation in the RAMOS system was conducted with three selected phosphate concentrations (Figure 3A). Derived from the results of Figure 2, a culture with 3000 mg/L dipotassium phosphate was chosen as reference for phosphate unlimited conditions. Besides that, cultivations with 150 mg/L and 75 mg/L K_2_HPO_4 _were conducted to represent intermediate and strong phosphate limitations, respectively. The unlimited culture showed a typical exponential growth reaching a maximal OTR of 55 mmol/L/h after 8 h. At this time, the OTR decreased sharply, showing a second small peak at 9 to 10 h. This behavior is typical for an *E.coli *batch cultivation where the primary carbon source, namely glucose, became exhausted (after 8 to 9 h) and the overflow metabolite acetate was consumed subsequently (after 9 to 11 h, Figure 3B). Although glucose, acetate and phosphate were not measured in shorter intervals, numerous studies dealing with the interpretation of OTR curves for microbial cultivations affirm this interpretation (e.g. [19,41,42]). Phosphate became not limited with 3000 mg/L K_2_HPO_4 _(Figure 3B). The OTR of the limited culture with 150 mg/L K_2_HPO_4 _started to stagnate at an OTR of 30 mmol/L/h after 6 to 7 h when phosphate limitation began (Figure 3C). After 11 h, the OTR dropped again, because glucose was exhausted. This culture showed a second short OTR plateau at about 12 h, mainly because of the metabolism of the hitherto produced acetate. The 75 mg/L-culture (Figure 3D) showed a similar pattern, although the OTR reached only 17 mmol/L/h at its maximum and slowly decreased until glucose was exhausted after about 15 to 17 h. This behavior is typical for a limitation of secondary substrates such as phosphate [19,28]. The culture started phosphate-limited growth after 5 to 6 h and showed a second small OTR plateau after 19 h, obviously because the residual amounts of acetate or other, not measured by-products were consumed. These findings, especially the times when phosphate limitation started and glucose was exhausted, are in good agreement with the growth data from the BioLector (Figure 2A).

The above results clearly showed that after a limitation of phosphate, the cells stagnated in growth and exhibited a prolonged respiration activity due to the ongoing consumption of the primary substrate (glucose) for maintenance. Similar results have been found with extensive offline analysis of growth, phosphate and glucose in a bioreactor [18,40]. The measurement of the respiration activity provided valueable insight into the metabolism of the cells and confirmed the proposed events during phoshate limitation. The period of a stagnating OTR depicted in Figure 3 (phosphate-limited cultures) represented a time of metabolic activity yet reduced growth of the cells and might be used to decouple recombinant protein expression from biomass growth.

### Effects of phosphate limitation on protein expression

**Figure 4:**
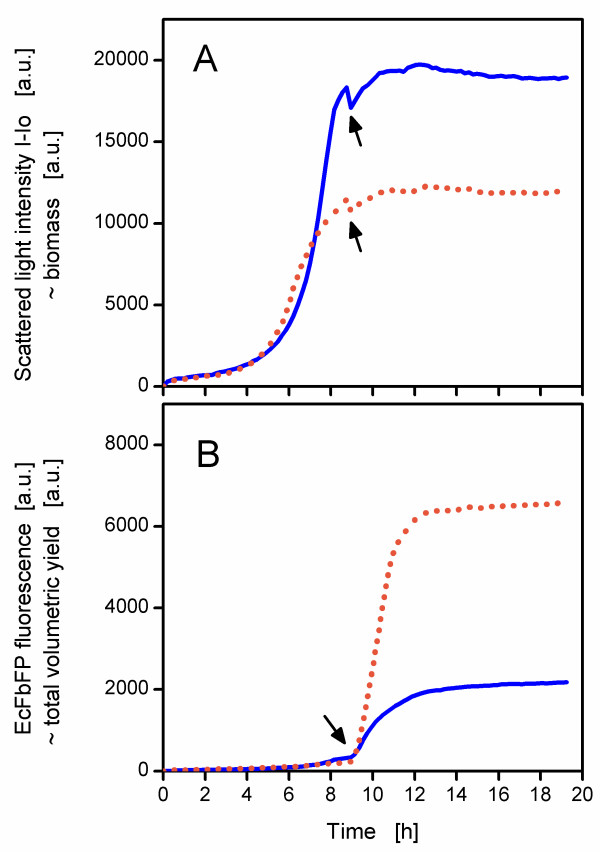
**Influence of phosphate limitation on growth (A) and recombinant protein production (B)**. The measurements (scattered light as an indicator for biomass, panel (A); EcFbFP fluorescence as an indicator for total volumetric yield, panel (B)) were conducted in the BioLector prototype with a signal gain factor of 25. Each of the four curves shown represent the mean values (n = 12) of cultures grown in media (modified Wilms & Reuss media with 7.5 g/L glucose) containing phosphate at two different concentrations: 3000 mg/L K_2_HPO_4 _(unlimited conditions, solid blue lines) and 150 mg/L K_2_HPO_4 _(phosphate-limited conditions, dotted orange lines). The relative standard deviation of each curve did not exceed ± 10%. The cultivation conditions were as follows: 37°C, shaking diameter 3 mm, shaking frequency 950 rpm, filling volume 200 μL, induction with IPTG (final concentration 0.1 mM) after 9.5 h (indicated by arrows).

A BioLector experiment was conducted to study in detail the general effects of a phosphate limitation on the expression of the fluorescence protein EcFbFP. Therefore, an intermediate phosphate concentration (150 mg/L) was chosen. Besides this, also the unlimited medium was used as a reference. From the results of Figure 2 and 3 an induction time of 9 to 10 h was chosen because at that time the phosphate became limited (for the 150 mg/L-culture). The unlimited 3000 mg/L-culture grew to a scattered light intensity of 19900 a.u. (values rounded to the nearest 100), whereas the 150 mg/L-culture reached only 13100 a.u. (Figure 4A). The unlimited culture immediately started to express the fluorescent protein EcFbFP upon induction and reached a plateau at a fluorescence of 2400 a.u. (Figure 4B).

After this culture consumed all the glucose, it reached stationary phase (13 h) and the cells had no longer the resources (substrate, energy) to express the target protein. On the other hand, the limited culture exhibited a much greater EcFbFP fluorescence (6900 a.u.) upon induction, even when the important nutrient phosphate was limited (Figure 4B). The production of EcFbFP continued until 14 h (probably because glucose was exhausted as shown in Figure 3 for an non-induced culture), though the cells did not grow anymore. Although a lower biomass was available in the phosphate-limited culture, even more EcFbFP was produced than in the unlimited culture. Accordingly, the specific productivity (EcFbFP fluorescence per scattered light intensity) at the end of the cultivation was higher for limited conditions (0.53 a.u.) than for the unlimited culture (0.12 a.u.). This provided evidence that phosphate-limited cells are still capable to produce recombinant proteins. The phosphate unlimited culture in this experiment was induced almost in the stationary phase and hence had only little resources left for efficient EcFbFP expression. Furthermore, the time of induction is probably the most crucial factor for recombinant protein expression [2]. Therefore, it is important to screen at different induction times for phosphate-limited and -unlimited cultures to evaluate if a phosphate limitation is generally suitable to enhance specific productivity.

For such a screening, a novel cultivation platform for automated high-throughput experimentation [32] was used. This system consists of a BioLector integrated into a liquid handling workstation. With this platform, it is possible to automatically induce up to 96 parallel cultures in a MTP at different times. This method, called 'induction profiling' [32], was modified here to test 9 different induction times (at a constant IPTG concentration of 0.1 mM) for phosphate-limited (150 mg/L) or unlimited cultures (3000 mg/L).

**Figure 5:**
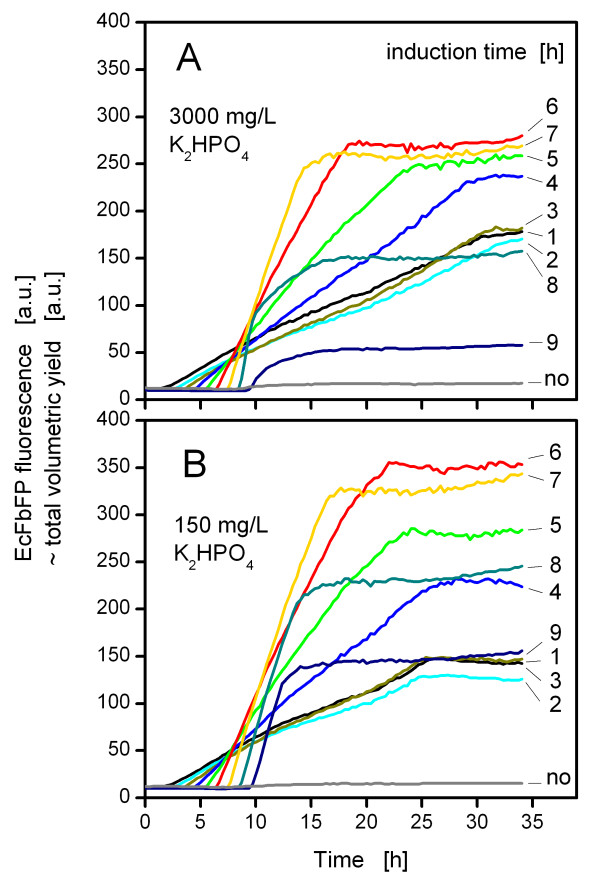
**Influence of induction time on formation of EcFbFP under (A) unlimited (3000 mg/L K_2_HPO_4_) and (B) limited (150 mg/L K_2_HPO_4_) conditions**. The measurements were performed with the Robo-Lector with a signal gain factor of 70. Each curve represents the EcFbFP production from duplicate cultures (n = 2) for every different time of induction. The relative standard deviation of each curve did not exceed ± 10%. The cultivation conditions were as follows: 37°C, shaking diameter 3 mm, shaking frequency 950 rpm, filling volume 200 μL, modified Wilms & Reuss medium (7.5 g/L Glucose), induction with IPTG (final concentration 0.1 mM) at 1 to 9 h.

**Figure 6:**
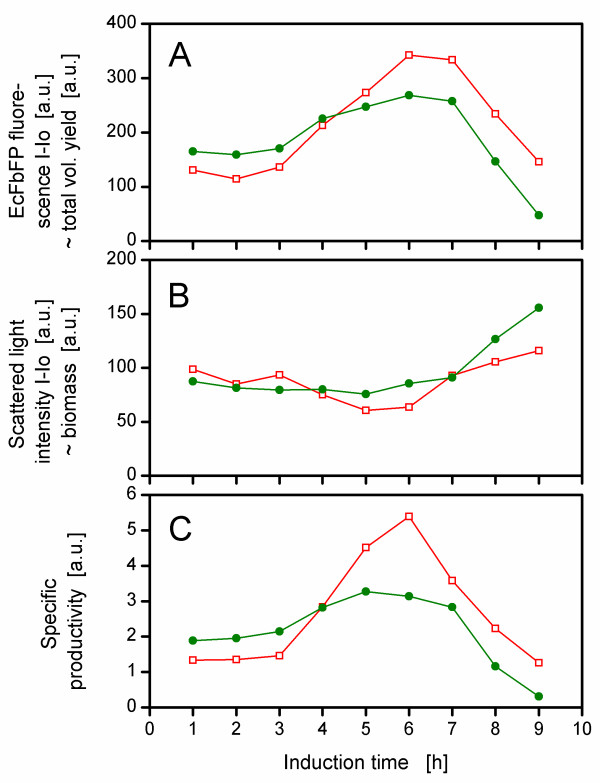
**Influence of induction time for phosphate-unlimited (3000 mg/L K_2_HPO_4_, •) and phosphate-limited conditions (150 mg/L K_2_HPO_4_, □) on (A) EcFbFP expression, (B) biomass yield and (C) specific productivity**. For experimental setup (growth conditions etc.), refer to Figure 5; data taken from experiment of Figure 5 at 34 h of cultivation.

Figure 5A depicts the EcFbFP expression in phosphate unlimited cultures at different induction times. As a reference, cells without induction were cultivated and showed only small background fluorescence. At every induction point (from 1 to 9 h after inoculation), an immediate increase of the EcFbFP fluorescence became visible for the different cultures. Noteworthy, the slope of this increase was steeper, the later the cells had been induced. This could be explained by the increased biomass at the later times of induction. The maximum EcFbFP production was achieved with an induction after 6 h. Afterwards, the protein expression decreased dramatically, probably because the medium became exhausted for glucose as already discussed for Figure 4. Similar effects resulted from inducing phosphate-limited cultures (Figure 5B), although here, in most cases, a higher EcFbFP fluorescence was observed than in unlimited cultures. To compare phosphate-unlimited and -limited conditions, the values (at 34 h) for the EcFbFP fluorescence at the different induction points were plotted in Figure 6A. The phosphate-limited and unlimited cultures displayed a nearly constant EcFbFP production until 3 h of induction time. After that, both phosphate conditions showed a peak in EcFbFP fluorescence at 6 h, whereas the phosphate-limited cultures reached higher values than the unlimited cultures. When cells were induced earlier than 4 h after inoculation, then the phosphate-limited cultures showed a lower EcFbFP expression. This could be explained by comparing the scattered light intensity with the specific productivity. Up to 4 h the biomass yield of both conditions was comparable (Figure 6B), though the specific productivity of the phosphate-limited culture was lower than for phosphate-unlimited conditions (Figure 6C), leading to a decreased overall expression (Figure 6A). After the 4 h induction point the specific productivity of the phosphate-limited cultures was significantly higher (Figure 6C) and together with only slightly less biomass (Figure 6B) resulted in more EcFbFP yield (Figure 6A).

In summary, the optimal time of induction could efficiently be determined with the applied experimental setup and revealed that the induction time was the predominant parameter for the overall expression yield. Furthermore it became obvious that phosphate limitation could only further enhance product formation when an appropiate induction time was chosen. These effects could easily be investigated with the Robo-Lector platform.

### Verification of results in shake flasks

**Figure 7:**
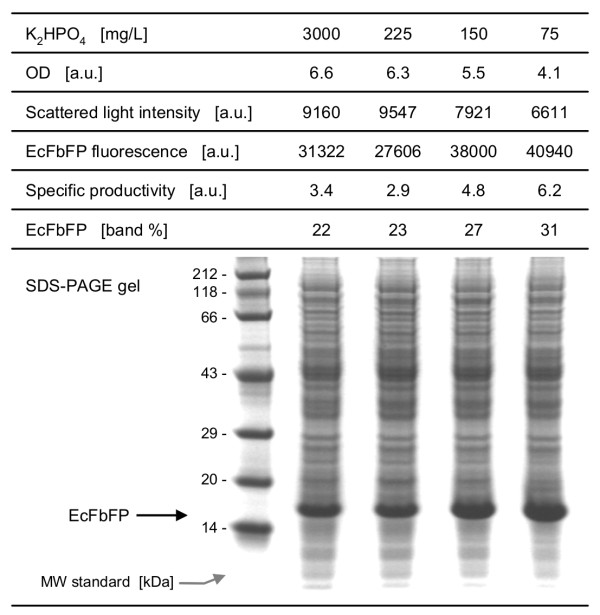
**Comparison of phosphate-limited and -unlimited conditions in shake flasks**. Comparison concerning biomass (OD and scattered light), product formation (EcFbFP fluorescence and SDS-PAGE gel) and specific productivity (fluorescence per scattered light and percent of EcFbFP band). Data from shake flask cultivations under the following conditions: 37°C, shaking diameter 50 mm, shaking frequency 350 rpm, filling volume 10 mL, modified Wilms & Reuss medium (7.5 g/L Glucose) with four different phosphate concentrations, induction with IPTG after 6 h (final concentration 0.1 mM), harvest time 26 h. Samples for SDS-PAGE were normalized to a comparable protein content and EcFbFP bands were quantified with densitometry; MW means molecular weight standard. Measurement of scattered light intensity (signal gain factor 20) and EcFbFP fluorescence (signal gain factor 45) were conducted with the BioLector prototype.

To verify the results from the previous experiments, a cultivation in shake flasks was conducted under different phosphate-limited (225 mg/L, 150 mg/L, 75 mg/L) and -unlimited conditions (3000 mg/L). The induction conditions were the same as in the Robo-Lector test with induction after 6 h, as this was the optimal time identified previously (Figure 6). After 26 h, samples from the different flasks were taken and the OD, scattered light and EcFbFP fluorescence was measured offline. Furthermore, samples were taken for SDS-PAGE and subsequent densitometric analysis. The results of these experiments were summarized in Figure 7.

The OD and scattered light intensity decreased with decreasing phosphate content of the medium (as could be expected from results of Figure 2). On the other hand, the EcFbFP fluorescence increased with decreasing phosphate, confirming the concept of increased product formation via a phosphate limitation in batch cultures (Figure 6). This was also indicated by the calculated specific productivity (EcFbFP fluorescence per scattered light), as this rose from 3.4 to 6.2 a.u. under phosphate-limited conditions. The percentage of the EcFbFP band per total protein content of the *E.coli *cells (band%), as determined with SDS-PAGE and densitometry, confirmed this finding (increase from 22 to 31%; see also SDS-PAGE gel).

## Conclusions

In this study, different online-monitoring tools, such as RAMOS and BioLector, were used to study, characterize and optimize the given T7 expression system *E.coli *BL21(DE3) pRhotHi-2-EcFbFP. With these tools, the demand of the examined strain for phosphate as well as the time of the limitation onset could easily be determined. Furthermore, the effect of phosphate limitation on target protein production and the optimal time for induction was efficiently investigated by the automated high-throughput cultivation platform Robo-Lector. Lastly, the results were sucessfully verified by shake flask experiments.

These results revealed, that the phosphate limitation was suitable to redirect the available resources during cultivation (after 'enough' biomass has formed) to protein expression rather than in biomass production. This effect finally led to an increase in specific productivity of the target protein EcFbFP from 21% to 31% of total protein. To our knowledge, such an effect was for the first time shown for an IPTG-inducible expression system. This is attributed to the fact that cells can be metabolically active for a long time even under a phosphate limitation and the resulting stationary phase [43]. This principle was verified with RAMOS cultivations shown in Figure 3. Han and Lee also showed by proteome analysis that a subset of proteins involved in protein synthesis in *E. coli *was greatly enlarged during a phosphate limitation [10]. This, in turn, could increase protein synthesis capacity. Additionaly, a higher plasmid copy number was observed in a non-induced fed-batch culture because of a phosphate limitation [44]. This might lead to higher amounts of mRNA for the target protein and consequently to a higher product yield in induced cultures. Furthermore, the aspects described here are most probably also the main reason why host/vector systems using the phosphate starvation-inducible promoter *phoA *show high productivities, as they tend to be very efficient expression systems [9,17].

Increasing the specific productivity by phosphate limitation can be especially important in high-throughput cultivations such as MTP-based processes. Since in MTPs the cultivation volume is mostly fixed and the biomass often can not be increased due to oxygen limitations, the only way to increase the total productivity is to raise the specific productivity [1]. A promising idea would also be to combine the presented online-monitoring tools with 'omics'-techniques to further investigate the described effects of phosphate limitation, as the exact molecular reasons for the increased specific productivity remain unclear. This could help to find new targets to further enhance the production capacity of recombinant *E.coli*-strains.

## Competing interests

The authors declare that they have no competing interests.

## Authors' contributions

RH designed and coordinated the study and prepared the manuscript. SR and NR performed cultivation experiments. JB assisted with data analysis and manuscript preparation. All authors read and approved the final manuscript.
